# The Activity of Metalloproteases and Serine Proteases in Various Organs after *Leiurus macroctenus* Envenomation

**DOI:** 10.1155/2023/5262729

**Published:** 2023-02-20

**Authors:** Valery Gunas, Oleksandr Maievskyi, Nataliia Raksha, Tetiana Vovk, Oleksiy Savchuk, Serhii Shchypanskyi, Igor Gunas

**Affiliations:** ^1^Department of Forensic Medicine and Law, National Pirogov Memorial Medical University, Pyrohova Street 56, Vinnytsya, Ukraine; ^2^Department of Clinical Medicine, Educational and Scientific Center “Institute of Biology and Medicine” of Taras Shevchenko National University of Kyiv, Hlushkova Avenue 2, Kyiv, Ukraine; ^3^Department of Biochemistry, Educational and Scientific Center “Institute of Biology and Medicine” of Taras Shevchenko National University of Kyiv, Hlushkova Avenue 2, Kyiv, Ukraine; ^4^Department of Human Anatomy, National Pirogov Memorial Medical University, Pyrohova Street 56, Vinnytsya, Ukraine

## Abstract

**Background:**

Scorpion stings may be life-threatening since their venoms are comprised of a wide range of toxins and other bioactive molecules, such as enzymes. At the same time, scorpion envenomation may increase matrix metalloproteases (MMPs) levels, which enhance proteolytic tissue destruction by venom. However, investigations on the impact of many scorpions' venoms, such as those of *Leiurus macroctenus*, on tissue proteolytic activity and MMP levels have not yet been conducted.

**Methods and Results:**

The present study aimed to examine the total proteolysis levels in various organs after *Leiurus macroctenus* envenomation and evaluate the metalloproteases and serine proteases' contributions to the total proteolytic activity. Changes in MMPs and TIMP-1 levels were tested as well. Envenomation led to a significant increase in proteolytic activity levels in all assessed organs, mostly in the heart (by 3.34 times) and lungs (by 2.25 times).

**Conclusions:**

Since EDTA presence showed a noticeable decrease in total proteolytic activity level, metalloproteases appeared to play a prominent role in total proteolytic activity. At the same time, MMPs and TIMP-1 levels were increased in all assessed organs, suggesting that *Leiurus macroctenus* envenomation causes systemic envenomation, which may induce multiple organ abnormalities, mostly because of the uncontrolled metalloprotease activity.

## 1. Introduction

Scorpions can be considered one of the most dangerous animals around the globe. Nearly 1.5 million envenoming cases are being reported annually, and this number may increase due to the rapid urban expansion [1]. Effects of venom on the human body vary from mild to severe and depend on a spectrum of parameters, but the most commonly reported symptoms are fever, nausea, dermatitis, hyperthermia, and haemorrhage, and without any treatment, envenomation leads to cardiac or respiratory failure [1, 2].

Scorpion-derived venom components have been identified and described [3]. Protein concentrations in scorpion venom can be relatively high. According to previous studies, proteins represent nearly half of *Leiurus abdullahbayrami* venom's dry weight [4]. The major components in scorpion's venom are toxins that act on potassium, sodium, calcium, and chloride channels (KTx, NaTx, CaTx, and ClTx, respectively), various enzymes (including serine proteases, metalloproteases, phospholipases, and hyaluronidases), protease inhibitors, and various peptides as well [1, 3, 5]. High levels of copper and zinc ions in *Leiurus quinquestriatus* venom also indicate the presence of various metalloproteases [6].

Once inside the body, venom components trigger the inflammatory response, which involves the liberation of various proinflammatory and anti-inflammatory cytokines and the attraction and activation of immune cells [7, 8]. Venom toxins block ion channels, causing paralysis, cardiac arrhythmia, and muscular spasms [9, 10]. At the same time, venom enzymes such as metalloproteases and serine proteases, phospholipases, and hyaluronidases are involved in toxin spreading via disintegration of matrix molecules; in addition, metalloproteases and serine proteases provide effective posttranslational processing of venom toxins [11, 12]. Venom presence also may increase levels of matrix metalloproteinases (MMPs), which act as inflammatory mediators [13] and enhance tissue damage. High levels of MMPs, whose concentrations are highly regulated by tissue inhibitors of metalloproteinases (TIMPs), also indicate high risks of cancer development because matrix destruction provides cell motility and allows cancer cells to invade extracellular matrix [14, 15].

Scorpions of the genus *Leiurus* (“deathstalker”) are extremely dangerous [16], but their venom can be used in medicine [17–19]. Although the genus *Leiurus* for a long time was considered monotypic, Lowe et al. have recently described a few new species, including *Leiurus macroctenus* [20], which can be distinguished from another species by morphometric and morphological characters [21].

To the best of our knowledge, there is no data about the impact of particular enzymes present in *Leiurus macroctenus* venom on proteolytic activity in tissues during envenomation or information about changes in tissue MMP levels after a *Leiurus macroctenus* sting.

The aim of this study was to examine the proteolysis levels in various organs after *Leiurus macroctenus* venom injection and evaluate serine proteases and metalloproteases' contributions to the total proteolytic activity. Furthermore, we tested the MMPs' content level changes as a response to envenomation.

## 2. Materials and Methods

### 2.1. Scorpion Collection and Maintenance

In this study, 10 mature *Leiurus macroctenus* specimens were used. All scorpions were previously identified by Mark Stockmann and kept in private collection in Ibbenbüren (Germany). Scorpions were maintained separately in transparent plastic boxes (10 × 5 × 5 cm) layered with sand (Exo Terra « Desert Sand») by 1 cm. In the center of each box were placed water bowls with distilled water, which were refilled every week. All animal containers were kept in constant conditions (25–35°C, 50–60% humidity, natural lighting conditions). Proper aeration was reached by numerous holes in container. Specimens were fed upon 1 *Shelfordella lateralis* cockroach once a week, in case of refusal of food, cockroach was taken away in 2 days after feeding. Once a month, containers were cleaned of cockroach remnants.

### 2.2. Venom Milking

The procedure of venom collection was performed according to Ozkan and Filazi's method [22], modified by Yaqoob [23]. Scorpion fixation was followed by electrodes pointing to the cephalothorax and telson. An electric current with an intensity of 24 V was applied for 5 seconds to the base of the telson, while the telson's opposite edge was pointed to the sterile phial. Depending on an amount of collected venom, number of electrode-scorpion contacts varied up to 10. The interval between milking acts was ranged at 2 weeks. The collected venom samples were stored at −20°C.

### 2.3. Venom Injection and Organ Homogenization

The group, consisting of 60 albino male rats (180 g ± 3 g), were injected intramuscularly with 0.5 mL venom solution (28.8 *μ*g/mL) (LD50 = 0.08 mg/kg), dissolved in saline solution (0.9%). The control group (13 rats) was injected with 0.5 ml saline solution (0.9%) alone. Organ isolation and homogenization were performed at 4°С, right after animal execution. Homogenization was conducted using 50 mM Tris-HCl (*pH* 7.4) buffer with 140 mM NaCl and 1 mM EDTA addition. The volume of buffer used was five times higher (in grams) than the mass of isolated organs. The crude homogenate was centrifuged at 600 g for 15 min, with further supernatant collection and its centrifugation at 15000 g for 15 min. Obtained homogenate aliquots were frozen in liquid nitrogen.

### 2.4. Total Proteolytic Activity Assessment

Total proteolytic activity was analyzed using the caseinolytic activity examination proposed by Kunitz [24] with some modifications. 4% casein solution was prepared by dissolving 4 g of casein in a mix of 80 ml 0.05 M phosphate buffer (*pH* 7.4) and 1.6 ml 1 M NaOH. The obtained mixture was left at room temperature for 40 min, and after that it was boiled in a hot water bath. The casein solution was cooled afterwards, *pH* 7.4 was adjusted by 1 M NaOH, and the total volume of 100 ml was adjusted by phosphate buffer. About 500 *μ*L of homogenate was diluted with 0.05 M phosphate buffer (*pH* 7.4) to a total volume of 1 mL. After that, 1 mL of 4% casein solution was added, and the obtained mixture was incubated at 37°C for 30 min. The reaction was stopped by a 3 mL addition of 15% TCA, after which samples were centrifuged at 1000 g for 30 min. The supernatant was collected and used for absorbance measurement at 280 nm. The control sample mixture consisted of casein, phosphate buffer, and TCA in identical proportions. In order to evaluate metalloproteases and serine proteases' activities, 0.2 M ethylenediaminetetraacetic acid (EDTA) and 0.2 M phenylmethylsulfonyl fluoride (PMSF), respectively were added to the reaction mixture. Total proteolytic activity was expressed as rel. units·(mg of protein)^−1^.

### 2.5. Quantification of Matrix Metalloproteases in Rat Organs Homogenates

Matrix metalloproteases quantification was conducted using immunoenzyme analysis standard method for soluble proteins [25]. The samples of homogenate were diluted to 1 *μ*g/mL with 0.05 М Tris-HCl buffer (*pH* 7.4) and incubated overnight at 4°С in sterile ELISA plates wells. After incubation, plates were washed with immobilization buffer and blocked for 1 hour at 37°С by 5% nonfat dry milk solution to avoid nonspecific binding. After that, plates were washed again with buffer containing 0.1% Tween-20, with further incubation with the corresponding primary antibodies (Santa Cruz Biotechnology, Inc., USA) (1 : 3000) for 1 hour at 37°С. Next, plates were washed with buffer containing 0.1% Tween-20 and incubated with HRP-conjugated secondary antibodies (Sigma-Aldrich, USA) (1 : 6000) for 1 hour at 37°С. After that, wells were washed with 0.1% Tween-20 buffer and incubated with 0.4 mg/ml o-phenylenediamine, diluted in 0.05 M citrate-phosphate buffer, in the presence of 30% Н_2_О_2_ to visualize the reaction. The reaction was stopped 10 minutes later by adding 100 *μ*L 1 М H_2_SO_4_. The optical density was measured at 492 nm using a microplate reader.

### 2.6. Protein Content Quantification

Protein contents were measured by Bradford assay [26].

### 2.7. Statistical Analysis

The values present in tables and figures are expressed as mean ± SEM. The significance of differences between the means of experimental groups was determined by one-way analysis of variances (ANOVA) with Tukey's multiple comparisons test, performed in GraphPad Prism 9. When ^∗^*p* < 0.05, ^∗∗^*p* < 0.005, ^∗∗∗^*p* < 0.001, and ^∗∗∗∗^*p* < 0.0001, differences between groups were considered statistically significant.

## 3. Results

### 3.1. Studies on Proteolytic Activity in Envenomation

**Figure 1:**
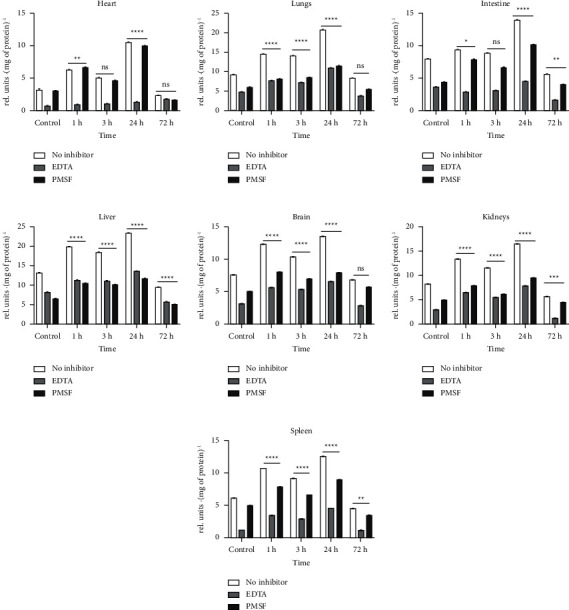
Total proteolytic activity levels after *Leiurus macroctenus* envenomation and influence of EDTA and PMSF on total proteolytic activity levels. Results are presented as mean ± SEM (*n* = 5). ^ns^*p* > 0.05, ^∗^*p* < 0.05, ^∗∗^*p* < 0.005, ^∗∗∗^*p* < 0.001, and ^∗∗∗∗^*p* < 0.0001 vs. control.

To examine proteolysis levels in various organs after *Leiurus macroctenus* venom injection and to evaluate metalloproteases and serine proteases' contributions, proteolytic activity in the presence and absence of ethylenediaminetetraacetic acid (EDTA) and phenylmethylsulfonyl fluoride (PMSF) was assessed. The results showed a considerable increment of total proteolytic activity after venom injection in all organs assessed; moreover, EDTA presence reduced proteolytic activity more than by 50% almost in all assessed organs (Figure 1). It is noticeable that an increase in proteolytic activity occurred in periods from the moment of venom injection to 1 hour after injection, and from the 3^rd^ hour after injection to 24 hours after injection. Minor decrements in proteolysis levels from the 1^st^ to the 3^rd^ hour of envenomation as well as major decrements from the 24^th^ to the 72^nd^ hour were observed.

Proteolysis level analysis in the heart showed a significant 3.34-fold increment of total proteolytic activity in 24 hours after venom injection, compared to the control (Figure 1(a)). In the presence of EDTA, total proteolytic activity decreased by 10% in 24 hours after venom injection. PMSF treatment showed a slight decrease in the proteolytic activity level of about 7% in 3 hours after venom injection.

According to Figure 1(b), the highest level of total proteolytic activity in the lungs was observed 24 hours after venom injection, a 2.25-fold increment compared to the control group. In contrast, with EDTA treatment, the proteolytic activity level remained unchanging during the experiment at about 51.8% of total proteolytic activity in inhibitor absence; nevertheless, in 72 hours after venom injection, it decreased by 7.3%. In the presence of PMSF, the highest proteolytic activity level decrement was observed in 24 hours after injection at about 45%.

A 1.75-fold increase in total proteolytic activity level in the intestine was also observed in 24 hours after envenomation (Figure 1(c)). In the presence of EDTA, total proteolytic activity was decreased by 15% in 1 hour after injection. In contrast, proteolytic activity in the presence of PMSF was relatively increased by 20% during envenomation.

As shown in Figure 1(d), in 24 hours after venom injection, the liver had a 1.77-fold increase in proteolytic activity level, compared to the control group. EDTA decreased the total proteolytic activity level by 5.2% in 1 hour after envenomation, while PMSF presence showed even higher rates of proteolytic activity during envenomation (51%–55%), compared to control (49%).

Based on Figure 1(e), the major increment of proteolytic activity level in the brain occurred in 24 hours after venom injection, about a 1.78-fold increment compared to the control group. EDTA and PMSF treatment led to an increase in proteolytic activity levels compared to the control values of 9.6% in 3 hours and 1.1% in 3 hours after injection, respectively.

Total proteolytic activity assessment in the kidneys showed a 2-fold proteolytic activity increment in 24 hours after venom injection (Figure 1(f)), compared to the control. EDTA treatment did not show a decrease in total proteolytic activity during envenomation; it increased by 10.2%, compared to the control value; nevertheless, PMSF in 3 hours after venom injection decreased the proteolytic activity level by 9%.

The results of total proteolytic activity analysis in the spleen showed a 2.04-fold increment in 24 hours after venom injection (Figure 1(g)), compared to the control group. An EDTA treatment resulted in 36.8% of total proteolytic activity level in 24 hours after venom injection, which is higher than the control value by 17%. At the same time, PMSF has decreased proteolytic activity level by 9.5% in 24 hours after venom injection.

### 3.2. MMPs and TIMP Levels Analysis

Taking into account the results from the evaluation of metalloproteases' contribution to proteolytic activity, an examination of the content levels of different types of matrix metalloproteases (MMPs) and tissue inhibitors of matrix metalloproteases (TIMPs) was performed. The results showed a prominent increase in levels of collagenases (MMP-1, MMP-8), gelatinases (MMP-2, MMP-9), stromelysins (MMP-3, MMP-10), and TIMP-1 in all organs assessed; moreover, peak levels were observed in the 24 hours after venom injection, with further decrement of MMP and TIMP levels approximately to initial ones (Table 1).

To sum up, total proteolytic activities as well as MMPs and TIMP-1 levels in the heart, lungs, intestine, liver, brain, kidneys, and spleen after *Leiurus macroctenus* envenomation were examined. As it turned out, *Leiurus macroctenus* envenomation causes significant elevations in proteolytic activity, mostly in the heart (3.34-fold increment) and lungs (2.25-fold increment). In the presence of EDTA, proteolytic activity was decreased by more than half in almost all organs, which indicates high metalloproteases content and activity during envenomation. In the presence of PMSF, proteolytic activity levels remained mostly above 60–70% in all organs, suggesting that serine proteases do not play a prominent role in proteolysis during envenomation. MMPs and TIMP-1 content assessments showed a considerable increase in levels of all MMPs and TIMP-1 in all organs assessed. Thus, the *Leiurus macroctenus* sting provides systemic envenomation, which may lead to multiple organ dysfunctions, mostly due to the uncontrolled metalloprotease activity.

## 4. Discussion

As one of the most dangerous creatures on Earth, scorpions can cause huge problems when they encounter humans. It was previously reported that systemic envenomation effects are mostly caused by autonomic nervous system abnormalities [27]. However, local and systemic inflammation due to venom proteases activity are no less harmful. Such uncontrolled proteolytic activity in various tissues may lead to severe consequences, such as necrosis and further organ dysfunction [28].

This study has revealed a significant increment in proteolytic activity levels during *Leiurus macroctenus* envenomation. Among all organs assessed, the heart and lungs appeared to have the highest increase in proteolytic activity after venom injection. Taking into account that cardiomyopathy and pulmonary edema are common symptoms of scorpion envenomation [29–32], protease activity may play a relevant role in the development of cardiovascular and respiratory system dysfunctions during *Leiurus macroctenus* envenomation.

According to the results obtained from the evaluation of metalloproteases and serine proteases' contributions to total proteolytic activity, metalloproteases were the major proteolytic agents in our experiment conditions, since EDTA treatment resulted in a reduction of the total proteolytic activity by more than half in most organs. A considerable decrement of proteolytic activity in EDTA presence during envenomation (as was shown for the heart, intestine, and liver) indicates an increase in total proteolytic activity due to the increase in metalloproteases levels. In the presence of constant proteolytic activity levels and a parallel rise in total proteolytic activity, as was shown for the lungs, metalloproteases' absolute levels rose proportionally to total proteolytic activity, while their relative levels remained the same. In the brain, kidneys, and spleen, EDTA has not decreased total proteolytic activity compared to the control, proving that in these organs total proteolytic activity increased mainly due to the increment of other proteases' activities. PMSF treatment did not significantly reduce total proteolytic activity; it remained above 60–70% in almost all organs, even though in the heart, lungs, kidneys, and spleen it was lightly decreased during envenomation, suggesting that serine proteases are minor participants in tissue proteolysis processes during envenomation. With PMSF, higher proteolytic activity levels after venom injection, as compared to the control, indicate a decrease in relative serine proteases content during envenomation. Other authors report that metalloproteases, which are responsible for severe envenomation symptoms, are commonly present in relatively high concentrations in some jellyfish [33] and snake [34] venoms, and the obtained results testify that *Leiurus macroctenus* venom is not an exception.

Taking into account the significant contribution of metalloproteases to the total proteolytic activity during envenomation, MMP and TIMP-1 levels were examined. The results showed a prominent increment of MMPs levels with a peak in 24 hours after venom injection in all organs assessed. Nevertheless, TIMP-1 levels were increasing simultaneously with MMP levels, thus, high TIMP-1 concentration decreased MMP levels in 72 hours after venom injection approximately to physiological values. The potential effects of MMP levels rising and their activation in tissues are varied, since there are several classes of MMPs based on their substrate type and structural properties [15]. Even though in different organs uncontrolled MMPs level increment leads to several diseases, basic effect is the same–degradation of extracellular matrix proteins and weakening of the matrix with further carcinogenesis [35]. Previous reports provided evidence about the role of MMP-1 and MMP-8, also known as collagenase 1 and collagenase 2, respectively, in the development of idiopathic pulmonary fibrosis [36], ischemic stroke, small vessel stroke [37], acute hepatitis [38], and various cancer types [39]. It was recently shown that the consequences of MMP-2 and MMP-9 (gelatinase A and gelatinase B, respectively) levels rising can be referred to as acute and chronic asthma, idiopathic pulmonary fibrosis [40], disruption of the blood-brain barrier [35], hepatic fibrosis (mainly due to MMP-2 overexpression) [41], and that these MMPs are the key molecules for tumor invasion [42] and congestive heart failure [43]. Stromelysins, namely MMP-3 and MMP-10, can be involved in wound healing, skeletal development, and other physiological processes. However, rapid and uncontrolled increases in their concentration and activity may lead to lung tumorigenesis [44], aortic valve calcification [45], idiopathic pulmonary fibrosis [46], Parkinson's and Alzheimer's diseases [47], rheumatoid arthritis [48], rheumatic heart disease and atherosclerosis [49]. TIMP-1 is one of the TIMPs, which mitigates most of the MMPs in a wide range of organs. Although TIMP-1 presence testifies MMP activity decrease, it can also be considered as a marker of several disease progressions [35].

Consequently, the *Leiurus macroctenus* sting may cause severe systemic envenomation since alterations in total proteolytic activity and MMP levels have been found in all organs assessed. The outcomes of envenomation may differ due to the large number of potential pathologies, which proteases, particularly metalloproteases, can trigger.

## Figures and Tables

**Table 1:** MMPs and TIMP levels after *Leiurus macroctenus* envenomation.

	Control	1 hour	3 hours	24 hours	72 hours
Heart, rel. units (g of tissue)^−1^					
MMP-1	108.2 ± 9.8	121.5 ± 5.6^∗∗∗^	133.5 ± 2.3^∗∗∗∗^	148.1 ± 4.7^∗∗∗∗^	116.3 ± 5.6^ns^
MMP-8	92.0 ± 6.9	103.4 ± 3.2^∗∗∗^	113.6 ± 5.9^∗∗∗∗^	126.1 ± 3.8^∗∗∗∗^	99.0 ± 8.1^ns^
MMP-2	244.3 ± 19.8	274.4 ± 7.8^∗∗∗^	301.5 ± 6.5^∗∗∗∗^	334.5 ± 7.6^∗∗∗∗^	262.6 ± 8.9^ns^
MMP-9	175.9 ± 4.6	197.7 ± 4.5^∗∗∗^	217.2 ± 7.8^∗∗∗∗^	241.0 ± 5.7^∗∗∗∗^	189.1 ± 5.6^ns^
MMP-3	211.7 ± 17.5	237.9 ± 8.9^∗∗∗^	261.4 ± 3.8^∗∗∗∗^	290.0 ± 4.9^∗∗∗∗^	227.6 ± 6.7^ns^
MMP-10	281.6 ± 12.4	316.4 ± 7.6^∗∗∗^	347.7 ± 9.5^∗∗∗∗^	385.8 ± 8.7^∗∗∗∗^	302.8 ± 5.2^ns^
TIMP-1	293.3 ± 15.4	329.5 ± 9.3^∗∗∗^	362.1 ± 5.7^∗∗∗∗^	401.8 ± 6.2^∗∗∗∗^	315.4 ± 9.8^ns^
Lungs, rel. units (g of tissue)^−1^					
MMP-1	216.9 ± 21.7	243.7 ± 4.7^∗^	267.8 ± 7.9^∗∗∗∗^	297.2 ± 4.9^∗∗∗∗^	233.2 ± 6.9^ns^
MMP-8	212.3 ± 24.6	238.6 ± 6.9^∗^	262.2 ± 8.1^∗∗∗∗^	290.9 ± 9.7^∗∗∗∗^	228.3 ± 5.9^ns^
MMP-2	130.5 ± 9.2	146.6 ± 9.8^∗^	161.1 ± 5.9^∗∗∗∗^	178.7 ± 9.8^∗∗∗∗^	140.3 ± 9.7^ns^
MMP-9	198.5 ± 14.7	223.1 ± 7.9^∗^	245.1 ± 2.9^∗∗∗∗^	272.0 ± 8.3^∗∗∗∗^	213.5 ± 7.8^ns^
MMP-3	205.6 ± 8.9	231.0 ± 8.7^∗^	253.8 ± 4.7^∗∗∗∗^	281.6 ± 9.6^∗∗∗∗^	221.1 ± 9.8^ns^
MMP-10	216.7 ± 8.8	243.5 ± 6.3^∗^	267.6 ± 5.7^∗∗∗∗^	296.9 ± 8.7^∗∗∗∗^	233.1 ± 6.9^ns^
TIMP-1	249.9 ± 42.0	280.8 ± 7.8^∗^	308.5 ± 6.9^∗∗∗∗^	342.3 ± 8.4^∗∗∗∗^	268.7 ± 7.8^ns^
Intestine, rel. units (g of tissue)^−1^					
MMP-1	110.3 ± 6.7	124.0 ± 4.5^∗∗∗∗^	136.2 ± 6.7^∗∗∗∗^	151.2 ± 5.6^∗∗∗∗^	118.6 ± 9.9^ns^
MMP-8	93.9 ± 3.9	105.5 ± 7.6^∗∗∗∗^	116.0 ± 5.7^∗∗∗∗^	128.7 ± 8.6^∗∗∗∗^	101.0 ± 8.4^ns^
MMP-2	249.2 ± 4.6	280.0 ± 7.8^∗∗∗∗^	307.7 ± 9.2^∗∗∗∗^	341.4 ± 7.5^∗∗∗∗^	267.9 ± 7.6^ns^
MMP-9	179.5 ± 7.8	201.7 ± 6.5^∗∗∗∗^	221.6 ± 7.5^∗∗∗∗^	245.9 ± 5.8^∗∗∗∗^	193.0 ± 7.8^ns^
MMP-3	216.0 ± 8.9	242.7 ± 9.5^∗∗∗∗^	266.7 ± 8.5^∗∗∗∗^	295.9 ± 9.3^∗∗∗∗^	232.3 ± 8.1^ns^
MMP-10	287.4 ± 8.9	322.9 ± 5.2^∗∗∗∗^	354.8 ± 8.1^∗∗∗∗^	393.7 ± 9.1^∗∗∗∗^	309.0 ± 5.7^ns^
TIMP-1	299.3 ± 6.1	336.3 ± 3.7^∗∗∗∗^	369.5 ± 2.6^∗∗∗∗^	410.0 ± 10.1^∗∗∗∗^	321.8 ± 6.2^ns^
Liver, rel. units (g of tissue)^−1^					
MMP-1	43.1 ± 2.5	48.5 ± 7.5^ns^	53.3 ± 6.9^∗^	59.1 ± 6.5^∗∗∗∗^	46.4 ± 5.4^ns^
MMP-8	41.6 ± 13.8	46.8 ± 8.1^ns^	51.4 ± 6.8^∗^	57.0 ± 9.2^∗∗∗∗^	44.8 ± 2.9^ns^
MMP-2	70.2 ± 15.7	78.8 ± 9.5^ns^	86.6 ± 7.8^∗^	96.1 ± 4.9^∗∗∗∗^	75.4 ± 6.8^ns^
MMP-9	89.5 ± 9.5	100.6 ± 4.9^ns^	110.5 ± 7.9^∗^	122.7 ± 5.4^∗∗∗∗^	96.3 ± 5.4^ns^
MMP-3	81.0 ± 11.1	91.1 ± 7.6^ns^	100.1 ± 2.9^∗^	111.0 ± 6.8^∗∗∗∗^	87.1 ± 7.9^ns^
MMP-10	111.5 ± 17.4	125.3 ± 7.7^ns^	137.7 ± 5.6^∗^	152.7 ± 7.6^∗∗∗∗^	119.9 ± 6.7^ns^
TIMP-1	81.4 ± 11.0	91.5 ± 5.6^ns^	100.5 ± 7.8^∗^	111.5 ± 7.1^∗∗∗∗^	87.5 ± 7.5^ns^
Brain, rel. units (g of tissue)^−1^					
MMP-1	233.2 ± 5.6	262.1 ± 4.7^∗∗∗∗^	288.0 ± 5.6^∗∗∗∗^	319.5 ± 4.5^∗∗∗∗^	250.8 ± 6.9^∗^
MMP-8	228.3 ± 9.7	256.5 ± 7.8^∗∗∗∗^	281.9 ± 9.3^∗∗∗∗^	312.8 ± 7.8^∗∗∗∗^	245.5 ± 6.9^∗^
MMP-2	140.3 ± 7.9	157.6 ± 8.3^∗∗∗∗^	173.2 ± 7.8^∗∗∗∗^	192.2 ± 8.1^∗∗∗∗^	150.8 ± 7.8^∗^
MMP-9	213.5 ± 8.1	239.8 ± 5.6^∗∗∗∗^	263.5 ± 6.7^∗∗∗∗^	292.4 ± 9.3^∗∗∗∗^	229.5 ± 7.7^∗^
MMP-3	221.1 ± 7.5	248.4 ± 9.2^∗∗∗∗^	272.9 ± 9.1^∗∗∗∗^	302.8 ± 9.1^∗∗∗∗^	237.7 ± 7.9^∗^
MMP-10	233.1 ± 6.8	261.9 ± 9.1^∗∗∗∗^	287.7 ± 7.7^∗∗∗∗^	319.3 ± 9.1^∗∗∗∗^	250.6 ± 6.2^∗^
TIMP-1	268.7 ± 7.9	301.9 ± 6.3^∗∗∗∗^	331.7 ± 8.8^∗∗∗∗^	368.1 ± 9.9^∗∗∗∗^	288.9 ± 4.5^∗^
Kidneys, rel. units (g of tissue)^−1^					
MMP-1	230.8 ± 6.9	259.3 ± 6.7^∗∗∗∗^	284.9 ± 5.9^∗∗∗∗^	316.1 ± 6.7^∗∗∗∗^	248.1 ± 3.9^∗∗^
MMP-8	225.9 ± 6.1	253.8 ± 2.3^∗∗∗∗^	278.9 ± 9.3^∗∗∗∗^	309.5 ± 6.3^∗∗∗∗^	242.9 ± 7.9^∗∗^
MMP-2	138.8 ± 8.7	155.9 ± 7.9^∗∗∗∗^	171.3 ± 7.8^∗∗∗∗^	190.1 ± 9.8^∗∗∗∗^	149.2 ± 6.8^∗∗^
MMP-9	211.2 ± 5.8	237.3 ± 4.8^∗∗∗∗^	260.7 ± 4.9^∗∗∗∗^	289.3 ± 6.9^∗∗∗∗^	227.1 ± 4.8^∗∗^
MMP-3	218.7 ± 3.9	245.7 ± 5.8^∗∗∗∗^	270.0 ± 7.5^∗∗∗∗^	299.6 ± 8.5^∗∗∗∗^	235.2 ± 5.6^∗∗^
MMP-10	230.6 ± 6.7	259.1 ± 6.7^∗∗∗∗^	284.7 ± 3.3^∗∗∗∗^	315.9 ± 5.2^∗∗∗∗^	247.9 ± 5.5^∗∗^
TIMP-1	265.8 ± 9.1	298.7 ± 9.7^∗∗∗∗^	328.2 ± 6.8^∗∗∗∗^	364.2 ± 5.9^∗∗∗∗^	285.8 ± 6.7^∗∗^
Spleen, rel. units (g of tissue)^−1^					
MMP-1	235.8 ± 2.9	264.9 ± 5.8^∗∗∗∗^	291.1 ± 6.8^∗∗∗∗^	323.0 ± 6.7^∗∗∗∗^	253.5 ± 6.7^∗∗^
MMP-8	230.8 ± 8.1	259.3 ± 6.8^∗∗∗∗^	285.0 ± 9.3^∗∗∗∗^	316.2 ± 9.8^∗∗∗∗^	248.2 ± 8.3^∗∗^
MMP-2	141.8 ± 9.8	159.3 ± 7.1^∗∗∗∗^	175.1 ± 5.8^∗∗∗∗^	194.3 ± 5.8^∗∗∗∗^	152.5 ± 7.9^∗∗^
MMP-9	215.8 ± 9.4	242.5 ± 9.1^∗∗∗∗^	266.4 ± 6.8^∗∗∗∗^	295.6 ± 5.2^∗∗∗∗^	232.0 ± 8.1^∗∗^
MMP-3	223.5 ± 6.8	251.1 ± 5.9^∗∗∗∗^	275.9 ± 4.9^∗∗∗∗^	306.1 ± 9.5^∗∗∗∗^	240.3 ± 5.6^∗∗^
MMP-10	235.6 ± 6.8	264.7 ± 5.9^∗∗∗∗^	290.9 ± 5.9^∗∗∗∗^	322.8 ± 6.7^∗∗∗∗^	253.3 ± 6.7^∗∗^
TIMP-1	271.6 ± 7.4	305.2 ± 6.7^∗∗∗∗^	335.3 ± 6.9^∗∗∗∗^	372.1 ± 4.3^∗∗∗∗^	292.1 ± 3.5^∗∗^

Notes: Results are presented as mean ± SEM (*n* = 5). ^ns^*p* > 0.05, ^∗^*p* < 0.05, ^∗∗^*p* < 0.005, ^∗∗∗^*p* < 0.001, and ^∗∗∗∗^*p* < 0.0001 vs. Control.

## Data Availability

The data used to support the findings of this study may be released upon application to Valery Gunas, who can be contacted by e-mail: forensic@vnmu.edu.ua; mob. +380669497912; or Pirogova 56 Street, Vinnytsia, Ukraine.
